# Impact of a provider-level incentive on smoking cessation treatment use: A secondary dataset analysis using administrative claims data and a cross-sectional survey in Japanese health check-up settings (N-EQUITY2203)

**DOI:** 10.18332/tid/218804

**Published:** 2026-05-09

**Authors:** Keiichi Yuwaki, Miyuki Odawara, Junko Saito, Maiko Fujimori, Aya Kuchiba, Manami Inoue, Koshiro Kanaoka, Taichi Shimazu

**Affiliations:** 1 Division of Behavioral Sciences, National Cancer Center Institute for Cancer Control, National Cancer Center, Tokyo, Japan; 2 Department of Cancer Epidemiology, Graduate School of Medicine, The University of Tokyo, Tokyo, Japan; 3 Graduate School of Public Health, Teikyo University, Tokyo, Japan; 4 Division of Survivorship Research, National Cancer Center Institute for Cancer Control, National Cancer Center, Tokyo, Japan; 5 Division of Biostatistical Research, National Cancer Center Institute for Cancer Control, National Cancer Center, Tokyo, Japan; 6 Division of Prevention, National Cancer Center Institute for Cancer Control, National Cancer Center, Tokyo, Japan; 7 Department of Medical and Health Information Management, Open Innovation Center, National Cerebral and Cardiovascular Center, Osaka, Japan

**Keywords:** smoking cessation, healthcare administrative claims, retrospective studies, cross-sectional studies, Japan

## Abstract

**INTRODUCTION:**

Healthcare professionals are recommended to deliver smoking cessation support (SCS) to smokers. However, the effectiveness of provider-level financial incentives on smoker’s behaviors, including the use of smoking cessation treatment (SCT), has been controversial. The aim of this observational study was to explore the association of a provider-level incentive to deliver a general lifestyle intervention, including SCS, with the smoker’s use of SCT, and describe the current state of and barriers to delivering SCS at health check-up settings.

**METHODS:**

This observational study in a health check-up setting consisted of: 1) a retrospective, quasi-experimental study through secondary use of an administrative claims database between 2018 and 2020; and 2) a cross-sectional questionnaire survey in 2023. Change in the proportion of SCT users pre- and post-introduction of the incentive was analyzed using a claims database, employing a difference-in-difference (DID) approach using adjusted linear regression. To describe SCS at health check-up settings, a cross-sectional survey was sent to 26 health check-up centers that had received the incentives which requested a response from a healthcare professional at each center. Responses were presented as descriptive statistics.

**RESULTS:**

DID analysis using data for 126137 smokers revealed no significant change in the proportion of SCT users following introduction of the provider-level incentive (DID= -0.15; 95% CI: -1.09–0.80 per 1000 smokers, p=0.759). The survey, which received responses from 24 health check-up centers, showed that only 27% of these centers provided smokers with contact information for SCT services. Furthermore, 71% and 67% of centers cited a lack of resources and self-efficacy, respectively, as perceived barriers to the delivery of SCS.

**CONCLUSIONS:**

The provider-level incentive to deliver the general lifestyle intervention was not associated with SCT use in health check-up settings. Increasing SCT use may require strategies aimed at overcoming barriers to the delivery of SCS.

## INTRODUCTION

Tobacco smoking is a leading risk factor for morbidity and mortality caused by non-communicable diseases (NCDs), including cancers, cardiovascular diseases, and chronic respiratory diseases^1,2^. Quitting smoking can lower the risk of developing NCDs among smokers^3^. In Japan, smoking cessation treatment (SCT) has been covered by the public health insurance system since 2006^4^, in accordance with the Framework Convention on Tobacco Control (FCTC), an evidence-based international treaty for tobacco control measures^5,6^. The SCT consists of five sessions with behavioral counseling and smoking cessation medications for 12 weeks. Subsequently, 60–80% of smokers achieve smoking cessation at the end of the SCT^7,8^. SCT use is an important step in achieving smoking cessation.

Healthcare professionals are recommended to deliver smoking cessation support (SCS) to every smoker during each healthcare site visit^9^. In Japan, the ABR method is recommended as SCS^10^. This method involves asking about tobacco use (‘Ask’), explaining the importance of smoking cessation and suggesting its solutions (‘Brief advice’), and referring smokers to evidence-based smoking cessation services, including SCT (‘Refer’). In lung cancer screening, delivery of the ABR method by public health nurses increased the abstinence rate by 3.29 times in comparison to usual care^10,11^. SCS functions at the population level as a gateway to SCT, an intensive individual approach to smoking cessation^6^.

Some countries, including Japan, have implemented health check-ups for the general population with the aim of preventing and screening diseases^12,13^. A cross-sectional survey in 2022 reported that 69% of Japanese adults undergo annual health check-ups^14^. These health check-ups represent an optimal setting for the delivery of SCS to large numbers of smokers, particularly those who have not yet developed NCDs. However, a previous review revealed that attending health check-ups was not associated with smoking cessation^15^. Increasing SCT use requires strategies that facilitate the implementation of appropriate SCS in health check-up settings.

One promising strategy to increase the delivery of SCS is financial incentives. A systematic review showed that provider-level incentives aimed at the SCS-related behaviors of providers, including smoking status recording and cessation advice provision, increased those behaviors in primary care settings^16^. Provider-level incentives are expected to improve smoker behaviors, such as SCT use or cessation achievement, through appropriate SCS. Nevertheless, the effectiveness of provider-level incentives on smoker behavior has been controversial^16,17^. The impact of provider-level financial incentives on smoker SCT use and barriers to the delivery of SCS in real-world health check-up settings should be evaluated.

Here, a retrospective, quasi-experimental, observational study was conducted to explore the association of a provider-level financial incentive to promote the delivery of a general lifestyle intervention, which included SCS but did not specify the required actions of SCS, with smokers’ use of SCT. Furthermore, the current state of and barriers to the delivery of SCS in health check-up settings were investigated.

## METHODS

### Study design

This observational study consisted of two studies. First, a retrospective, quasi-experimental, observational study through secondary use of an administrative claims database was conducted in the health check-up setting between 2018 and 2020. The study aimed to explore the association of a program offering financial incentives to providers to deliver the lifestyle intervention at health check-up settings with SCT use by using a difference-in-difference (DID) approach. The administrative claims database included health check-up results and insurance claim data of individuals insured by the Japan Health Insurance Association (JHIA). The JHIA is the largest health insurance association for workers in Japan and covers employees of medium-sized or smaller companies and their dependents aged less than 75 years^13,18^.

Second, a cross-sectional questionnaire survey on SCS in health check-up settings was conducted. The survey was conducted among healthcare professionals working at health check-up centers to determine the extent to which SCS has been implemented. The survey’s findings were then used to interpret the results of the DID analysis. Additionally, the survey investigated barriers that must be overcome for SCS delivery.

The study, including the retrospective analysis through secondary use of an administrative claims database and the cross-sectional questionnaire survey, was approved by the National Cancer Center Institutional Review Board (No. 2022-261).

### Setting

Employees insured by JHIA and aged ≥35 years are subsidized for general health check-ups once in a fiscal year (FY), which starts in April and ends in March in Japan. In FY 2018, the Shizuoka branch of JHIA introduced an incentive program that aimed to support health check-up centers in the implementation of a general lifestyle intervention. The purpose of the lifestyle intervention was to increase interest in health among insured employees who received health check-ups and encourage them to seek medical treatment, including SCT, if necessary. The intervention was delivered by healthcare professionals working at health check-up centers, namely public health nurses, registered nurses, and registered dietitians, within a five-minute period on the day that the insured employee visited the center. Although a brief instructional document was available that outlined the purpose, target participants, and providers of the lifestyle intervention, the document did not specify the detailed actions of providers, such as the referral to SCT. As a result, the conduct of the lifestyle intervention was determined by each health check-up center, and the SCS for smokers lacked standardization. As a financial incentive, each health check-up center was paid 637 Japanese yen (about US$4) per insured employee who received the lifestyle intervention. The Shizuoka branch recruited health check-up centers for the incentive program. A total of 5, 16, and 5 health check-up centers participated in FYs 2018, 2019, and 2020, respectively.

### Retrospective analysis through secondary use of the administrative claims database

*Study population*

This study was based on the secondary use of an administrative claims database of employees insured by the Shizuoka branch of JHIA between April 2013 and March 2021, i.e. FYs 2013 to 2020. The database is classified as anonymously processed information, and the requirement for informed consent was accordingly waived.

Employees in the original administrative claims database were categorized by FY, namely from FYs 2014 to 2019 (Supplementary file Figure 1). For example, an employee who was insured from October 2014 to December 2016 was included in the subject population of FYs 2014, 2015, and 2016. The subjects in each FY comprised insured employees who met the following inclusion criteria: 1) individuals who received a health check-up subsidized by JHIA during this FY; 2) individuals aged 35–74 years at the health check-up; 3) individuals who responded that they were current smokers in the health check-up questionnaire; 4) individuals who had been insured for at least 12 months prior to and 3 months following the health check-up; and 5) individuals who had not visited a clinic for SCT in the 12 months prior to the health check-up.

Health check-up centers participating in the incentive program in FY 2018, i.e. the first FY that the Shizuoka branch of the JHIA introduced this program, might have already delivered lifestyle interventions, including SCS, prior to FY 2018. To evaluate the impact of the incentive program on delivery of the lifestyle intervention, insured employees who received the health check-up at a participating center in FY 2018 were excluded. Consequently, the change between FYs 2018 (pre-introduction period) and 2019 (post-introduction period) among subjects who visited a participating health check-up center in FY 2019 (incentive group) was compared to the change among the other subjects (control group) using a DID approach.

*Variables*

The original database contained information on demographic characteristics, including sex, age, size of the employer company, and monthly income; results of the health check-up, including body mass index, waist circumference, blood pressure, and blood test results; responses to the self-reported questionnaire in the health check-up, including current smoking status and medication use, past medical history, and stage of lifestyle improvement based on the Transtheoretical model^19^; status of receipt of specific health guidance; and health insurance claims for medical service fees for the SCT. In addition, the database included the FY in which the health check-up center participated in the incentive program.

*Outcomes*

The primary outcome was the proportion of SCT users. Secondary outcomes were the proportion of SCT completers, the proportion of quitters, and smoking prevalence in the next year. These outcomes were evaluated using the data of subjects of FYs 2018 and 2019. The proportion of SCT users was defined as the number of smokers who used the SCT within three months after their health check-up per 1000 smokers. The proportion of SCT completers was defined as the number of smokers who completed the SCT within four months after their first visit to the SCT per 1000 smokers. The use and completion of the SCT based on health insurance claims is defined in the Supplementary file. The Supplementary file also provides details on the proportion of quitters, defined as the number of smokers who achieved quitting in the next year per 100 smokers, and smoking prevalence in the next year, defined as the number of smokers who responded as a current smoker in the next year per 100 subjects.

*Statistical analysis*

The association between the introduction of the incentive program and the outcomes changes was examined using a DID approach to control for background trends involving smoking cessation^20^. The differences in outcome between the pre- and post-introduction periods in the incentive group, compared with the control group, were evaluated.

Baseline characteristics of the incentive and control groups were described together with the number and proportion of subjects in each category in both the pre- and post-introduction periods. The differences in outcomes were calculated by subtracting the outcomes in the pre-introduction period from the outcomes in the post-introduction period. The DID estimates were calculated using unadjusted and adjusted linear regression models. These estimates were obtained from the interaction term between the groups (incentive or control groups) and the periods (pre- and post-introduction periods). Potential covariates were adjusted in two models (details in the Supplementary file). The parallel trend assumption was assessed through both visual and statistical inspection (details in the Supplementary file). The common shock assumption was supported, given that JHIA did not deliver other smoking cessation interventions in FY 2019. All analyses were performed using R^21^.

### Cross-sectional survey

*Study population*

In March 2023, an invitation to participate in an online questionnaire survey was sent to all health check-up centers that met the eligibility criteria, defined as entry into the incentive program between FYs 2018 and 2020 and continuation in it through FY 2022 (n=26). No additional exclusion criteria were applied. A healthcare professional responsible for the lifestyle intervention at each health check-up center was designated as the respondent regarding the overall status of SCS delivery at the center and barriers to its delivery, and gave consent before answering the questionnaire survey.

*Questionnaire*

A question on SCS delivery was developed based on the components of the ABR method^10^. The question asked healthcare professionals whether they delivered components of the ABR method and additional support to smokers in the lifestyle intervention. A question on barriers to delivering SCS at the health check-up setting was developed based on previous studies^22-24^ and consultation with healthcare professionals (details in the Supplementary file). The question asked about ten barriers, including lack of self-efficacy or knowledge, and was mapped to four domains of the Consolidated Framework for Implementation Research (CFIR), with the exception of the implementation process domain^25,26^ (Supplementary Table 1). The perception of potential barriers was assessed using a 5-point Likert scale (‘strongly agree’, ‘agree’, ‘neither agree nor disagree’, ‘disagree’, and ‘strongly disagree’).

*Data analysis*

Responses to the questionnaire are presented as descriptive statistics, with the number and proportion of respondents in each question. With regard to the barriers to delivering SCS, responses of ‘strongly agree’ and ‘agree’ were classified as actual barriers perceived by healthcare professionals.

## RESULTS

### Retrospective analysis through secondary use of the administrative claims database

Table 1 shows the baseline characteristics of the 126137 subjects stratified by the control group (45223 in pre- and 45928 in post-introduction periods) and incentive group (17639 in pre- and 17347 in post-introduction periods) in both periods. In both groups across both periods, approximately 40% of subjects were aged 40–49 years, and more than 80% of subjects were male.

**Table 1:** Baseline characteristics of smokers included in the difference-in-differences analysis before and after introduction of the provider-level incentive program, Japan Health Insurance Association administrative claims data, Shizuoka Prefecture, fiscal years 2018–2019 (N=126137)

*Characteristics*	*Pre-introduction period (FY2018)*		*Post-introduction period (FY2019)*	
	*Control group* *(N=45223)* *n (%)*	*Incentive group* *(N=17639)* *n (%)*	*Control group* *(N=45928)* *n (%)*	*Incentive group* *(N=17347)* *n (%)*
**Age** (years)				
35–39	7340 (16.2)	2604 (14.8)	7173 (15.6)	2505 (14.4)
40–49	18255 (40.4)	7271 (41.2)	18392 (40.0)	6952 (40.1)
50–59	12560 (27.8)	4943 (28.0)	13031 (28.4)	4986 (28.7)
60–69	6394 (14.1)	2518 (14.3)	6553 (14.3)	2552 (14.7)
70–74	674 (1.5)	303 (1.7)	779 (1.7)	352 (2.0)
**Sex**				
Male	37296 (82.5)	14616 (82.9)	37638 (82.0)	14328 (82.6)
**Company size** (number of employees)				
<10	8997 (19.9)	4280 (24.3)	8777 (19.1)	4156 (24.0)
10–29	9668 (21.4)	4442 (25.2)	10160 (22.1)	4611 (26.6)
30–49	5306 (11.7)	2123 (12.0)	5444 (11.9)	2212 (12.8)
50–99	6243 (13.8)	2714 (15.4)	6486 (14.1)	2631 (15.2)
100–299	7233 (16.0)	2067 (11.7)	7431 (16.2)	1966 (11.3)
300–499	1996 (4.4)	877 (5.0)	2044 (4.5)	894 (5.2)
500–999	2039 (4.5)	903 (5.1)	2011 (4.4)	614 (3.5)
≥1000	3741 (8.3)	233 (1.3)	3575 (7.8)	263 (1.5)
**Monthly income** (JPY)				
<100000	233 (0.5)	133 (0.8)	222 (0.5)	136 (0.8)
100000–199999	5228 (11.6)	1905 (10.8)	5108 (11.1)	1752 (10.1)
200000–299999	12621 (27.9)	4943 (28.0)	12872 (28.0)	4834 (27.9)
300000–399999	15520 (34.3)	6110 (34.6)	15735 (34.3)	5989 (34.5)
≥400000	11621 (25.7)	4548 (25.8)	11991 (26.1)	4636 (26.7)
**Metabolic syndrome** (MetS)				
Individuals with MetS	11508 (25.4)	4429 (25.1)	11811 (25.7)	4471 (25.8)
**Medication**				
Diabetes	2196 (4.9)	850 (4.8)	2403 (5.2)	900 (5.2)
Dyslipidemia	3162 (7.0)	1118 (6.3)	3504 (7.6)	1227 (7.1)
Hypertension	5948 (13.2)	2201 (12.5)	6300 (13.7)	2257 (13.0)
**Past history**				
Cerebrovascular disease	371 (0.8)	144 (0.8)	407 (0.9)	157 (0.9)
Cardiovascular disease	844 (1.9)	333 (1.9)	895 (1.9)	329 (1.9)
Renal failure or dialysis	129 (0.3)	32 (0.2)	142 (0.3)	44 (0.3)
**Stage of lifestyle improvement**				
Precontemplation	15461 (34.2)	5553 (31.5)	15718 (34.2)	5358 (30.9)
Contemplation	16022 (35.4)	6851 (38.8)	16192 (35.3)	6784 (39.1)
Preparation	5494 (12.1)	2708 (15.4)	5710 (12.4)	2569 (14.8)
Action	2966 (6.6)	1179 (6.7)	3216 (7.0)	1240 (7.1)
Maintenance	2984 (6.6)	1308 (7.4)	3080 (6.7)	1359 (7.8)
**Specific health guidance** (SHG)				
Individuals who received SHG	1261 (2.8)	892 (5.1)	1369 (3.0)	983 (5.7)

Individuals with MetS: Those with a waist circumference ≥85 cm for men and ≥90 cm for women, or body mass index ≥25 kg/m^2^, accompanied by at least one of the following: 1) impaired glucose tolerance (blood glucose ≥100 mg/dL or HbA1c ≥5.6%); 2) dyslipidemia (triglycerides ≥150 mg/dL or high-density lipoprotein cholesterol <40 mg/dL); or 3) elevated blood pressure (systolic blood pressure ≥130 mmHg or diastolic blood pressure ≥85 mmHg). Individuals who had previously received medication for diabetes, dyslipidemia, or hypertension were excluded. DID: difference-in-difference. FY: fiscal year. JPY: 1000 Japanese Yen about US$6.3.

Table 2 shows changes in the proportion of SCT users and completers before and after the introduction of the incentive program. In both the control and incentive groups, the proportion of smokers who received SCT within three months following their health check-up was minimal, at one or two individuals per 1000 smokers. According to DID estimates calculated by a linear regression model, no significant change was found in proportion of SCT users (DID= -0.15; 95% CI: -1.09–0.80 per 1000 smokers, p=0.759) and completers (DID= -0.29; 95% CI: -1.06–0.49 per 1000 smokers, p=0.471) by the introduction of the incentive program. In addition, as presented in Supplementary Table 2, no significant changes were observed in the proportion of quitters (DID=0.01; 95% CI: -6.38–6.39 per 100 smokers, p=0.998) or in smoking prevalence in the next year (DID= -0.22; 95% CI: -6.57–6.12 per 100 smokers, p=0.946).

**Table 2:** Change in the proportion of smoking cessation treatment users and completers per 1000 smokers before and after introduction of the provider-level incentive program, Japan Health Insurance Association administrative claims data, Shizuoka Prefecture, fiscal years 2018–2019 (N=126137)

	*Pre-introduction period (FY 2018)*	*Post- introduction period (FY 2019)*	*Difference (Post - Pre)*	*Difference-in-Difference*					
				*Unadjusted Model*		*Adjusted Model 1*		*Adjusted Model 2*	
				*DID (95% CI)*	*p*	*DID (95% CI)*	*p*	*DID (95% CI)*	*p*
**Proportion of SCT users**									
Control group (ref.)	1.53	1.35	-0.18						
Incentive group	1.70	1.38	-0.32	-0.14 (-1.08–0.80)	0.769	-0.14 (-1.09–0.80)	0.769	-0.15 (-1.09–0.80)	0.759
**Proportion of SCT completers**									
Control group (ref.)	1.02	0.91	-0.10						
Incentive group	1.25	0.86	-0.38	-0.28 (-1.06–0.50)	0.480	- 0.28 (-1.05–0.50)	0.481	- 0.29 (-1.06–0.49)	0.471

DID: difference-in-difference. FY: fiscal year. SCT: smoking cessation treatment. Adjusted Model 1: sex and age. Adjusted Model 2: sex, age, number of employees in the company in which they were employed, monthly income, status of metabolic syndrome, medication use for diabetes, medication use for dyslipidemia, medication use for hypertension, past history of cerebrovascular disease, past history of cardiovascular disease, past history of renal failure or dialysis, stage of lifestyle improvement, and status of specific health guidance.

### Cross-sectional survey

**Figure 1:**
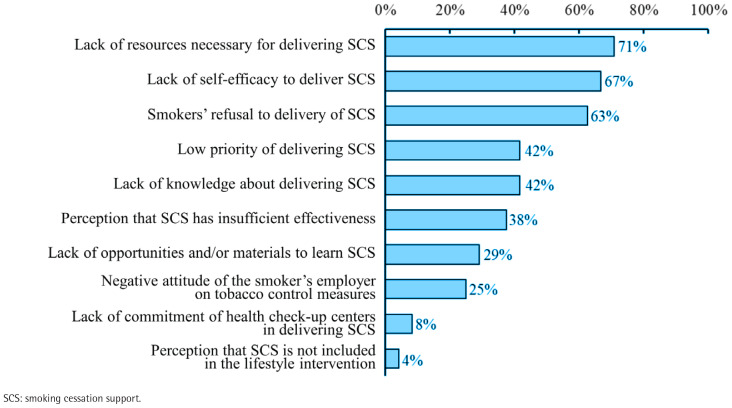
Proportion of health check-up centers reporting specific barriers to smoking cessation support delivery in a questionnaire survey, Shizuoka Prefecture, Japan, 2023 (N=24)

Twenty-four of the 26 health check-up centers responded to the questionnaire (response rate 92.3%). The components of the ABR method were delivered in 22 centers. While components of Ask and Brief advice were delivered in over 60% of health check-up centers, only 27% of centers provided smokers with contact information on SCT services (Table 3). The following barriers to SCS delivery were recognized by more than half of the health check-up centers surveyed: a lack of resources (proportion of healthcare professionals who selected, 71%; CFIR domain, inner setting), including personnel, time, and materials; a lack of self-efficacy (67%; individuals); and smoker refusal (63%; individuals) (Figure 1).

**Table 3:** Number of health check-up centers delivering each component of the ABR method among centers reporting implementation of the ABR method in a questionnaire survey, Shizuoka Prefecture, Japan, 2023 (N=22)

*Component*	*n (%)*
**Ask**	
Ask about tobacco use	22 (100)
Assess level of smoker’s intention to quit	18 (82)
**Brief advice**	
Communicate the need to quit	14 (64)
Emphasize that smoking cessation is an important and high-priority health issue	15 (68)
Provide information on the harm of smoking and benefit of smoking cessation tailored to the smoker’s situation	20 (91)
Provide information on easy, reliable, and less expensive solutions, such as SCT and medications	17 (77)
**Refer**	
Recommend smokers to visit SCT and/or medications	15 (68)
Provide smokers with contact information for SCT services	6 (27)
**Additional support (not included in the ABR method)**	
Make an appointment for SCT	0 (0)
Set a quit date	2 (9)
Listen to smoker’s concerns and worries about quitting and discuss its solutions	13 (59)

ABR: Ask-Brief advice-Refer. SCT: smoking cessation treatment.

## DISCUSSION

To our knowledge, this is the first study to explore the association of a provider-level financial incentive for delivery of a general lifestyle intervention, which included SCS but did not specify the required actions of SCS, in a real-world health check-up setting with SCT use through retrospective DID analysis by secondary use of a large-scale administrative claims database. Introduction of the incentive program aimed at implementing the general lifestyle intervention in health check-up settings, did not result in an increase in SCT use. Furthermore, interpretation of the results of the DID analysis based on a cross-sectional questionnaire survey of healthcare professionals working in health check-up centers revealed that while >60% of centers asked smokers about tobacco use and advised smoking cessation, only 27% provided contact information for SCT services. Additionally, the centers cited a lack of resources and self-efficacy, as well as smokers’ refusal, as barriers to SCS delivery. The findings of this study will contribute to the development of strategies for implementing SCS in health check-up settings.

The financial incentive provided to health check-up centers to deliver the general lifestyle intervention was not associated with smokers’ SCT use. Further, fidelity to the ABR method, especially the provision of contact information for SCT, was inadequate. Of note, healthcare professionals perceived multilevel barriers to SCS delivery, consistent with the findings of a previous study^27^. A non-specific financial incentive program for the delivery of the general lifestyle intervention without the specification of SCS actions, such as a structured referral pathway, might not be an effective strategy to overcome multilevel barriers to the delivery of appropriate SCS in health check-up settings. Consequently, it is hypothesized that SCS was not integrated into the standardized flow of health check-ups, which resulted in low SCT uptake. FCTC Article 14 mandates the implementation of effective measures to promote tobacco cessation as a population-level approach, including SCS in health check-up settings, and the provision of adequate treatment for tobacco dependence as an intensive individual approach, including SCT^5,6^. Despite the potential for health check-up settings to reach large numbers of smokers and provide SCS, they remain largely unutilized in this role. Implementation strategies that facilitate the appropriate SCS in health check-up settings are needed to increase SCT use. While the optimal incentive amount remains to be determined, increasing the incentive amount per single SCS delivery with limited financial resources is difficult. Furthermore, additional strategies to overcome the multilevel barriers to integrating SCS into health check-up settings are essential. For example, educational meetings and the development of educational materials may resolve the issue of a lack of self-efficacy, which was a barrier in the individual domain of the CFIR^28^. A previous study showed that healthcare professionals who participated in an interactive educational program on SCS, delivered SCS more frequently than before their participation^29^.

Among smokers who underwent a health check-up, only 0.1–0.2% received SCT within three months following the health check-up. A previous study based on the national health insurance database of Japan also revealed that approximately 1% of smokers received SCT annually in FY 2015^30^. These results indicated that the uptake of evidence-based SCT^7,8^ is limited in Japan. A similar situation has been documented in other countries. For example, a cross-sectional survey in the United States in 2014 and 2015 showed that only 5.5% of smokers had used prescribed smoking cessation medication in the past 12 months^31^. The cross-sectional survey revealed that only a limited number of health check-up centers provided contact information on SCT, and none of the centers actively arranged appointments for SCT. This failure to implement referral to SCT might result in a low SCT uptake. Addressing the evidence-practice gap on SCT use requires the implementation of SCS that reliably guides smokers toward SCT, which is consistent with the guidance for FCTC Article 14^6^. For instance, one promising approach to increasing SCT uptake is proactive referral, in which the healthcare professional actively connects the smoker to SCT^32^.

### Strengths and limitations

In this study, real-world evidence and several suggestions on SCS in health check-up settings were obtained from administrative claims data and a cross-sectional questionnaire survey. In addition to these strengths, several limitations also warrant mention. First, it was confirmed that JHIA did not provide smoking cessation interventions other than the incentive program. However, the implementation of worksite-level interventions was unknown. The implementation of different smoking cessation interventions across worksites may disrupt a common shock assumption. The results of the DID analysis might be biased toward the null if such interventions were provided only for subjects in the control group. However, the medium-sized and smaller companies where the subjects of our DID analysis worked, are less likely to implement such interventions^33^. Second, the extent to which the ABR method was implemented was reported by healthcare professionals in the questionnaire survey. Such self-reporting data may be subject to social desirability bias^34^, and the proportion of delivery of ABR components might accordingly have been overestimated. Third, subjects for the DID analysis were also defined using self-reported smoking status. Smoking prevalence based on self-reporting was lower than that determined by cotinine measurements in biological specimens^35^. Misclassification might therefore have occurred, such as the exclusion of smokers from the DID analysis or the underestimation of smoking prevalence. Fourth, the anonymized administrative claims database could not be linked to the response to the questionnaire survey, and statistical exploration of the associations between the incentive program and the provider’s SCS delivery and between its delivery and the smoker’s behaviors was therefore impossible. Fifth, subjects of the DID analysis were mainly employees working in Shizuoka Prefecture, Japan. The health check-up centers were also located in this area. The generalizability of the findings might therefore be limited to other geographical areas, particularly countries lacking universal health coverage or annual health check-ups. However, the findings may also be informative for countries with an organized health check-up system or workplace-based health promotion programs.

## CONCLUSIONS

This DID analysis of an administrative claims database found no association between a provider-level financial incentive program for delivery of a general lifestyle intervention, which included SCS but did not specify the required actions of SCS, in health check-ups with the use of SCT services. The cross-sectional questionnaire survey revealed that only a limited number of health check-up centers provided contact information on SCT as part of SCS. Furthermore, many centers cited a lack of resources and self-efficacy as barriers to the delivery of SCS in health check-up settings. The findings indicate that strategies to overcome these barriers to integrating SCS into the standardized flow of health check-ups may be necessary to increase SCT uptake.

## Supplementary Material



## Data Availability

The data supporting this research are available from the authors on reasonable request.
