# Supplementary material for: Impact of a provider-level incentive on smoking cessation treatment use: A secondary dataset analysis using administrative claims data and a cross-sectional survey in Japanese health check-up settings (N-EQUITY2203)
Source: Tob Induc Dis. 2026 May 9;24:10.18332/tid/218804. doi: 10.18332/tid/218804 (PMC13156808; doi:10.18332/tid/218804)
Supplement: Supplementary file 1 [file TID-24-57-s1.pdf]

Supplementary Methods

Definition of the use and completion of smoking cessation treatment

Clinics can charge "nicotine dependence management fees" for providing the smoking cessation treatment (SCT). Until March 2020, the fees consisted of three types of per-visit fee, namely a first-visit fee, a second-to-fourth-visit fee, and a fifth-visit fee. These per-visit fees are charged for each of the five sessions of the SCT. Consequently, SCT use and completion could be captured from claims of the first-visit fee and the fifth-visit fee, respectively. After April 2020, a per-series fee was added to the nicotine dependence management fees. This novel fee could be charged only once at the first visit to SCT. Therefore, if clinics charged a per-series fee, SCT use could be captured, but not SCT completion. Clinics can arbitrarily select charging either the per-visit fees or the per-series fee.

Details on the proportion of quitters and smoking prevalence in the next year

To analyze the proportion of quitters, individuals who did not respond to the question on their current smoking status in the next year, i.e., in FY 2019 for subjects of FY 2018 or in FY 2020 for subjects of FY 2019, were excluded. The proportion of quitters was defined as the number of smokers who achieved quitting in the next year per 100 smokers. Subjects who did not answer that they were current smokers in the health check-up questionnaire in the next year were classified as having achieved quitting.

To analyze smoking prevalence in the next year, non-smokers were included as subjects whereas individuals who did not respond to the question on their current smoking status in the next year were excluded. Smoking prevalence was defined as the number of smokers who responded as a current smoker in the next year per 100

subjects.

Potential covariates adjusted in DID analysis

Potential covariates were adjusted in two models. Model 1 was adjusted for sex (male or female) and age (35-39, 40-49, 50-59, 60-69, or 70-74 years). Model 2 was adjusted for sex; age; number of employees in the company in which they were employed (<10, 10-29, 30-49, 50-99, 100-299, 300-499, 500-999, or 1000≤); monthly income (<100000, 100000-199999, 200000-399999, or 400000≤ Japanese yen); status of metabolic syndrome (with or without); medication use for diabetes (with or without); medication use for dyslipidemia (with or without); medication use for hypertension (with or without); past history of cerebrovascular disease (presence, absence, or unknown); past history of cardiovascular disease (presence, absence, or unknown); past history of renal failure or dialysis (presence, absence, or unknown); stage of lifestyle improvement based on the Transtheoretical model [1] (precontemplation, contemplation, preparation, action, maintenance, or unknown); and status of specific health guidance (received or not).

Parallel trend assumption

The parallel trend assumption was assessed by examining the annual trends of outcomes between FYs 2014 and 2018 through visual inspection (Appendix Figure 2). Furthermore, an evaluation of the interaction term between groups (incentive and control groups) and periods (FYs 2014, 2015, 2016, 2017, and 2018) in the adjusted linear regression model 2 indicated that the parallel trend assumption was satisfied (proportion of SCT users, $p = 0.988$; proportion of SCT completers, $p = 0.778$;

proportion of quitters, $p = 0.104$; smoking prevalence, $p = 0.334$).

Development of a question on barriers

A question on barriers to the delivery of smoking cessation support (SCS) in health check-up settings was developed based on previous studies and consultation with healthcare professionals. Initially, the researchers (KY, ST) identified potential barriers based on several previous studies [2–4]. They then consulted with four healthcare professionals working in the Shizuoka branch of the JHIA (one public health nurse) and health check-up centers (two public health nurses and one registered dietitian) to confirm the question.

Reference

1. Prochaska JO, Velicer WF. The Transtheoretical Model of Health Behavior Change. *American Journal of Health Promotion*. 1997;12(1):38-48. doi:10.4278/0890-1171-12.1.38
2. Keyworth C, Epton T, Goldthorpe J, Calam R, Armitage CJ. Delivering Opportunistic Behavior Change Interventions: a Systematic Review of Systematic Reviews. *Prevention Science*. 2020;21(3):319-331. doi:10.1007/s11121-020-01087-6
3. Thornberry A, Garcia TJ, Peck J, Sefcik E. Occupational Health Nurses' Self-Efficacy in Smoking Cessation Interventions: An Integrative Review of the Literature. *Workplace Health Saf*. 2020;68(11):533-543. doi:10.1177/2165079920925106
4. Manolios E, Sibeoni J, Teixeira M, Révah-Levy A, Verneuil L, Jovic L. When primary care providers and smokers meet: a systematic review and metasynthesis. *NPJ Prim Care Respir Med*. 2021;31(1):31. doi:10.1038/s41533-021-00245-9

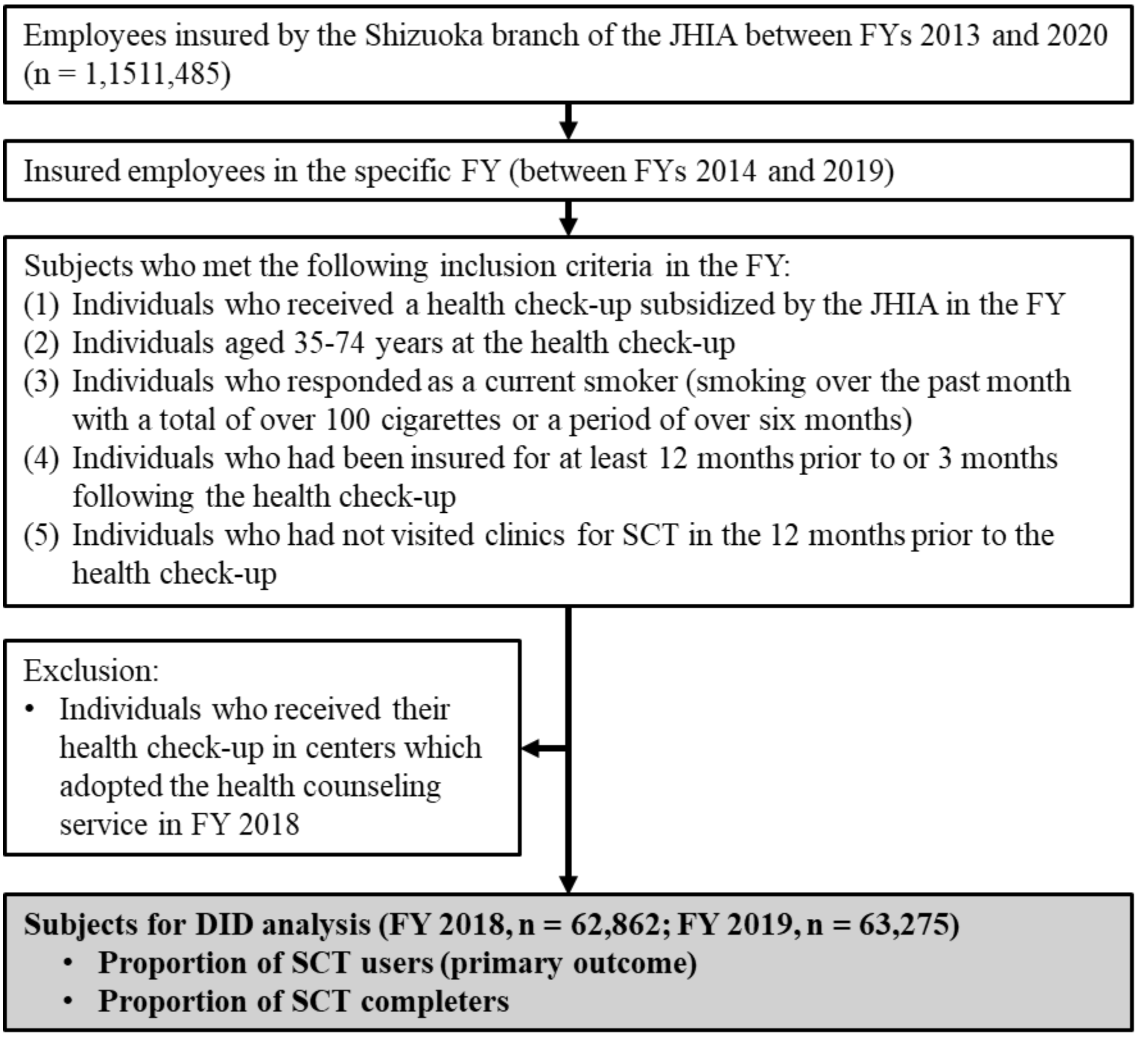

Supplementary Figure 1. Flow of subject selection for the difference-in-differences analysis before and after introduction of the provider-level incentive program, Japan Health Insurance Association administrative claims data, Shizuoka Prefecture, fiscal years 2013-2020 (N = 11511485).

DID, difference-in-difference; FY, fiscal year; JHIA, Japan Health Insurance Association; SCT, smoking cessation treatment

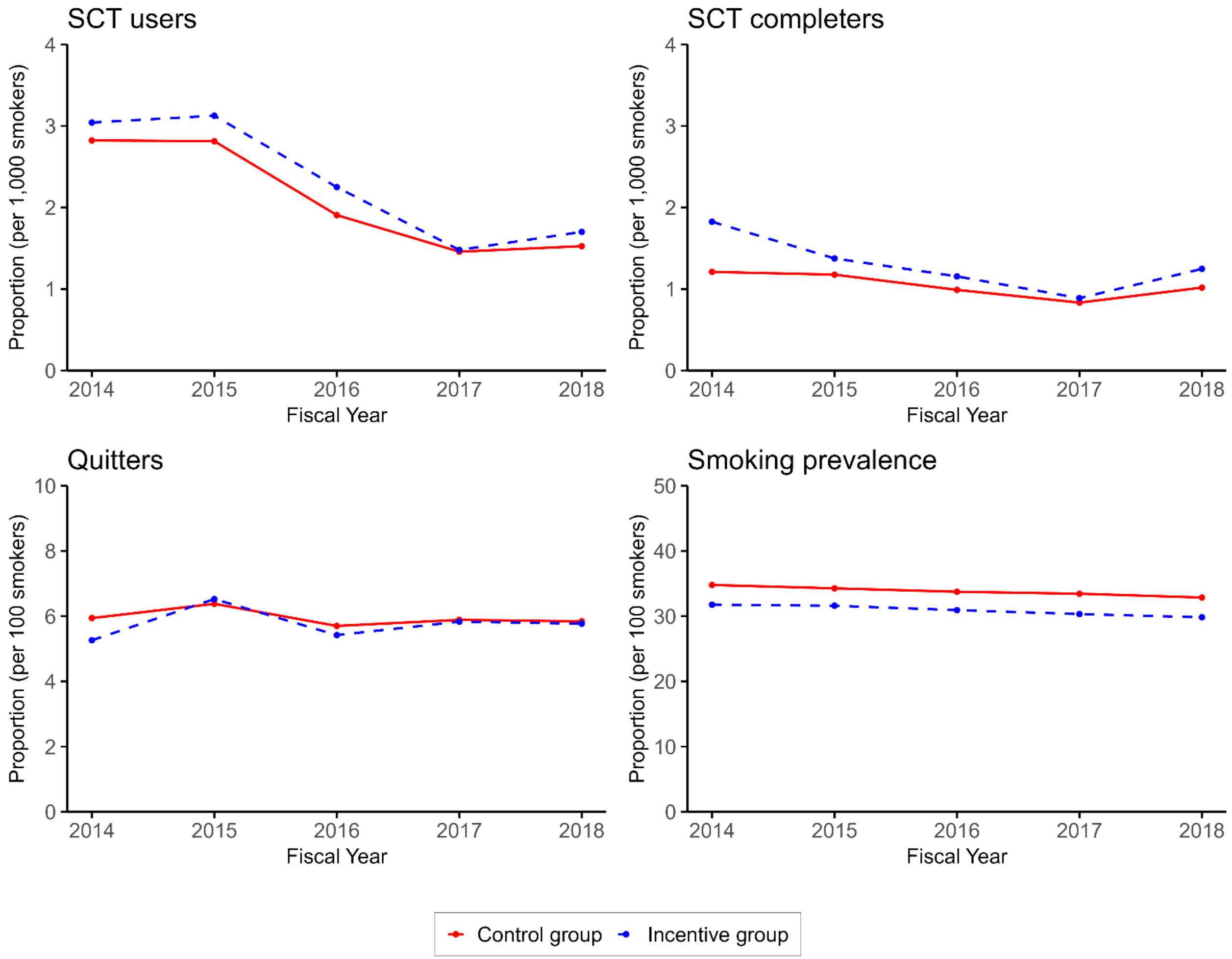

Supplementary Figure 2. Annual trends in outcomes on visual inspection of the parallel trend assumption for the difference-in-differences analysis before and after introduction of the provider-level incentive program, Japan Health Insurance Association administrative claims data, Shizuoka Prefecture, fiscal years 2014-2018 (N = 804021).

SCT, smoking cessation treatment.

Supplementary Table 1. Potential barriers to the delivery of SCS presented in the questionnaire for health check-up centers in Shizuoka Prefecture, Japan, 2023.

Potential barrier	Question ("To what extent do you agree that the following sentences are reasons for not delivering SCS through the health counseling service?")	CFIR domain	CFIR construct
Perception that SCS has insufficient effectiveness	SCS cannot lead smokers to achieve quitting.	Innovation<br>Individuals	Innovation Evidence-Base<br>Innovation Delivers - Motivation
Negative attitude of the smoker's employer on tobacco control measures	The employer of smokers receiving the health counseling service is reluctant to implement tobacco control measures.	Outer Setting	Local attitudes
Lack of commitment of health check-up centers in delivering SCS	My health check-up center is not committed to delivering SCS.	Inner Setting	Culture
Low priority of delivering SCS	SCS's priority is low, as our focus should be on other interventions.	Inner Setting	Relative Priority
Perception that SCS is not included in the lifestyle intervention	SCS is not included in the scope of the health counseling service.	Inner Setting<br>Individuals	Mission Alignment<br>Innovation Delivers - Motivation
Lack of resources necessary for delivering SCS	Resources necessary for delivering SCS, such as personnel, time, and materials, are insufficient.	Inner Setting	Available Resources
Lack of opportunities and/or materials to learn SCS	I cannot access opportunities and/or materials to learn SCS.	Inner Setting	Access to knowledge & Information
Lack of self-efficacy to deliver SCS	I am not confident in my capacity to deliver SCS.	Individuals	Innovation Delivers - Capability
Lack of knowledge about delivering SCS	I do not know how to deliver SCS.	Individuals	Innovation Delivers - Capability
Smokers' refusal to the delivery of SCS	Smokers do not request or accept receipt of SCS.	Individuals	Innovation Recipients - Need

Respondents chose from five options: “Strongly agree”, “Agree”, “Neither agree nor disagree”, “Disagree”, and “Strongly disagree”.
CFIR, Consolidated Framework for Implementation Research; SCS, smoking cessation support

Supplementary Table 2. Change in the proportion of quitters and smoking prevalence per 100 smokers before and after introduction of the provider-level incentive program, Japan Health Insurance Association administrative claims data, Shizuoka Prefecture, fiscal years 2018–2019 (N = 355671).

	Pre-introduction period (FY 2018)	Post-introduction period (FY 2019)	Difference (Post - Pre)	Difference-in-Difference					
				Unadjusted model		Adjusted model 1		Adjusted model 2	
				DID (95% CI)	p value	DID (95% CI)	p value	DID (95% CI)	p value
Proportion of quitters									
Control group	5.84	6.23	0.39	Ref		Ref		Ref	
Incentive group	5.77	6.20	0.43	0.03 (-0.61, 0.67)	0.921	0.02 (-0.62, 0..66)	0.949	0.00 (-0.64, 0.64)	0.998
Smoking prevalence									
Control group	32.86	32.22	-0.64	Ref		Ref		Ref	
Incentive group	29.83	29.00	-0.83	-0.19 (-0.86, 0.48)	0.570	-0.07 (-0.71, 0.57)	0.824	-0.02 (-0.66, 0.61)	0.946

CI, confidence interval; DID, difference-in-difference; FY, fiscal year.
Adjusted model 1: sex and age.
Adjusted model 2: sex, age, number of employees in the company in which they were employed, monthly income, status of metabolic syndrome, medication use for diabetes, medication use for dyslipidemia, medication use for hypertension, past history of cerebrovascular disease, past history of cardiovascular disease, past history of renal failure or dialysis, stage of lifestyle improvement, and status of specific health guidance.

© 2026 Yuwaki K. et al.
